# Maxillary Sinus Infections of Nasal and Dental Origin from the Standpoint of a Rhinologist*An address delivered before the Scientific Section of Oral Surgery, First District Dental Society, New York, March 21, 1923.

**Published:** 1923-10

**Authors:** Cornelius G. Coakley

**Affiliations:** New York, N. Y.


### MAXILLARY SINUS INFECTIONS OF NASAL AND DENTAL ORIGIN FROM THE STANDPOINT OF A RHINOLOGIST *

CORNELIUS G. COAKLEY, M. D., NEW YORK, N. Y.

(This summary is neither authentic nor complete. It represents the impression made by the address upon one in the audience.)

Infection in sinus cases arises from two separate sources: extension from the nasal mucous membrane to the accessory sinuses and from the buccal through wounds or, more frequently, through infection around the roots of the teeth or alveolar process. You, as dentists, and we, as rhinologists, probably differ as to the percentage of cases arising from each of these causes, more especially as figures compiled before our present methods of diagnosis were perfected are of little value. Dr. Babcock found that in 109 cases in private practice only 7½% could be ascribed to buccal origin. In 176 cases between October 1, 1922, and January 1, 1923, only five could be ascribed to buccal origin. Dentists would probably see a higher percentage of cases arising from buccal causes.

---

* An address delivered before the Scientific Section of Oral Surgery, First District Dental Society, New York, March 21, 1923.

The symptoms of maxillary sinusitis differ in acute and chronic cases. Acute cases sometimes exhibit pain, but not always. There is always a discharge from the nose. Except in cases of dental origin, deformity, such as swelling of the face, is rare.

Chronic cases often do not exhibit pain. They complain of a discharge from the nose or into the pharynx. About 50% of such cases that come to us have no idea that they have any involvement of the sinus. It is only under routine examination that we are able to decide which patients have maxillary sinusitis.

The diagnosis of maxillary sinusitis is comparatively simple if gone about in the right way. A large percentage of cases will be seen to have considerable secretion from the nose, either mucoid or purulent. However, if the patient blows the nose just before sitting in the chair for examination, it is quite possible that the person looking into the nose may be deceived by the absence of secretion. After cocainizing the patient, we examine the interior of the nose with the pharyngoscope. It is astonishing to note how often the use of this instrument will detect pus around the orifices of the frontal, maxillary or sphenoid sinus when the ordinary nasal speculum fails to show any pus. The absence of pus does not prove that the patient is free from sinusitis, because the cavity may contain a considerable amount of pus; but, if it is not overflowing when the case is examined, the nose and the region of the orifices from the frontal and maxillary sinuses may be free from pus. The pharyngoscope is especially useful in cases presenting a thin, purulent secretion.

The next step in examination is transillumination, which is quite simple and can be readily done by the oral surgeon. It requires only a dark room, a battery of three cells for the lamp and a proper lamp with a shield. The lamp, with the shield in place, should be put into the mouth and the brilliancy of the illumination through the sinus noted. This method is most valuable when only one sinus is involved, because the difference in the transillumination between the normal sinus and infected sinus is great. The benefits of this method of transillumination are so great that I hope it will become a part of your routine procedure in examining such cases.

When both of the sinuses are involved, the danger of being fooled by transillumination is great. It is important to know about how much illumination one can expect in a face of a given individual with normal antra, and this takes experience.

The next step in examination is to have radiographs of the skull taken antero-posteriorly. Such a radiograph is most valuable in determining whether there is a proper degree of illumination in the maxillary sinus. Other important things to be considered are whether or not you have a single or double sinusitis, who is taking the radiograph, the angle at which it is taken and the age of the patient. In an adult, with a large sinus, well aerated, the picture will be clear. In children, with less development, the shadows formed may easily be mistaken for diseased condition. It is difficult to get clear plates for reading, but plates which are not clear should not be regarded as diagnostic.

In cases of doubt, we have no hesitation in irrigating the antrum to find out what is there. In cases of blond, pale patients, it is surprising how much material may be in the maxillary sinus and yet the transillumination be good. To irrigate the maxillary sinus, we cocainize the naso-antral wall and pass a trocar through it with the aid of a small mallet, which causes less discomfort to the patient than hand pressure. The sinus is then irrigated with normal salt solution, and the fluid which passes out is caught in a black pus basin, which shows up the character of the fluid much better than a white basin. In a normal maxillary sinus, the fluid coming out in this way will be absolutely clear. In the early stages of acute maxillary sinusitis we have found almost clear mucous, nearly white and gelatinous. Twenty-four hours later, the secretion shows some yellow. In another day, it may be distinctly orange-yellow or of a greenish color. The color depends upon the associated bacteria. If there is Staphylococcus aureus, the color will be yellow. In acute cases the secretion sticks in one mass and the mucous, is of the color characteristic of the associated bacteria. Sometimes the irrigating fluid from the maxillary sinus looks like an emulsion because of the presence of broken-down purulent secretion, the product of a breaking-down action by some bacteria other than that which caused the disease. Odor is usually present only in chronic cases where putrefactive organisms have got into the note and so into the sinus. This condition may obtain with any of the sinuses. The presence of an odor might cause the rhinologist to investigate the possible dental cause for the sinusitis, but it does not follow that because the odor is present, the cause is dental. When odor is present in acute cases, we almost invariably find the cause of the sinusitis to be dental.

In acute cases, there is only an acute oedema of the membrane. If such a case can be relieved of the secretion and ventilated, it will probably get well in a short time. In cases which have been standing for months or years, serious pathological changes have taken place. Our method of treating acute cases is to wash out the secretion, to keep the nasal membrane clean, to watch each day and to note the diminishing amount of secretion and the improvement under transillumination.

The speaker said that he has no patience with those who advocated the use of suction as a means of drawing secretion from the sinus because it would not work in cases of thin secretion, though it might work in certain cases where the secretion held tenaciously together.

The antero-posterior radiographs, taken for purposes of general diagnosis, do not give information about the condition around the roots of the teeth. If there is reason to be suspicious of any tooth, each tooth which approaches the sinus must be radiographed. In a number of cases we have made no progress in treatment of the disease so long as there was a focus of infection about the roots of the teeth. With the removal of the infection about the teeth, the cases cleared up.

It is probable that in many acute cases of oral origin, the extraction of certain teeth, the passing of a probe of the proper number into the sinus and a few irrigations will result in a cure. Long-standing cases can not be successfully treated in that way because of the pathological changes in the membrane which are so extensive that a few treatments can not get the membrane back to a non-secreting condition. When a large opening is made from the mouth into the sinus, it permits the entrance of infective and putrefactive organisms and affords a source of constant infection, which does away with the chance of curing that case.

We have had several cases where dentists have made devices to close up an opening into the antrum. Sometimes the opening is closed by a tube and sometimes by means of a tooth. The tooth can not keep infection out of the antrum, and the tube drains only that secretion above the level of its upper end and leaves the residual secretion. The presence of any mechanical device in an opening of this sort results in the formation of an epithelized opening from the mouth to the sinus. That opening will remain and the inflammatory process will keep up. Such procedures are not good surgery.

In any long-standing case which can not be cleared up in four or five weeks, it is better to make a large opening into the antro-nasal wall under local anesthesia, taking off the anterior half of the inferior turbinate body. If the hole is left only one-half or three-quarters inch in diameter antero-posteriorly, it will not remain long enough for the thick membrane to return to normal, and the patient is then as badly off as before. Such an opening does not tend to cause reinfection, because the interior of the nose is practically free from bacteria, and the few present are generally destroyed by the action of the mucus. An antrum which has been infected is more susceptible to reinfection, when the patient gets a fresh cold, than a normal antrum would be.

Under such treatment, the patient either gets well or has a moderately thickened membrane, which is all that he can expect if the membrane has been thickened for years. Under the old method, when we packed an antrum for several months and let it fill up with granulation tissue, that tissue might become infected, and it required an operation to relieve it.

I do not know what is the best treatment of acute sinusitis of dental origin. Dentists believe in a small opening following the extraction of the tooth. This is not a good method in chronic cases, because the patient can not get well before an epithelized canal from the mouth to the antrum is established. This leaves the antrum more susceptible to reinfection than the opening from the nose.—*Dental Digest.*

